# Diurnal Intermittent Fasting during *Ramadan*: The Effects on Leptin and Ghrelin Levels

**DOI:** 10.1371/journal.pone.0092214

**Published:** 2014-03-17

**Authors:** Mohammed A. Alzoghaibi, Seithikurippu R. Pandi-Perumal, Munir M. Sharif, Ahmed S. BaHammam

**Affiliations:** 1 Department of Physiology, College of Medicine, King Saud University, Riyadh, Saudi Arabia; 2 Center for Healthful Behavior Change, Division of Health and Behavior, Department of Population Health, New York University Langone Medical Center, Clinical and Translational Research Institute, New York, New York, United States of America; 3 Somnogen Canada Inc., Toronto, Ontario, Canada; 4 The University Sleep Disorders Center, Department of Medicine, College of Medicine, King Saud University, Riyadh, Saudi Arabia; 5 National Plan for Science and Technology, King Saud University, Riyadh, Saudi Arabia; Monash University, Australia

## Abstract

We aimed to assess the effect of Islamic intermittent fasting, during and outside of *Ramadan*, on plasma levels of leptin and ghrelin while controlling for several potential confounding variables. Eight healthy male volunteers with a mean age of 26.6±4.9 years reported to the sleep disorders center (SDC) at King Saud University on four occasions: 1) adaptation; 2) 4 weeks before *Ramadan* while performing Islamic fasting for 1 week (baseline fasting) (BLF); 3) 1 week before *Ramadan* (non-fasting baseline) (BL); and 4) during the second week of *Ramadan* while fasting. Plasma leptin and ghrelin levels were measured using enzyme-linked immunoassays at 22:00, 02:00, 04:00, 06:00, and 11:00. During BLF, there were significant reductions in plasma leptin concentrations at 22:00 and 02:00 compared with the baseline concentrations (at 22:00: 194.2±177.2 vs. 146.7±174.5; at 02:00: 203.8±189.5 vs. 168.1±178.1; p<0.05). During *Ramadan*, there was a significant reduction in plasma leptin levels at 22:00 (194.2±177.2 vs. 132.6±130.4, p<0.05). No significant difference in plasma ghrelin concentrations was detected during the BL, BLF, or *Ramadan* periods. Cosinor analyses of leptin and ghrelin plasma levels revealed no significant changes in the acrophases of the hormones during the three periods. The nocturnal reduction in plasma leptin levels during fasting may be the result of the changes in *meal time*s during fasting.

## Introduction

*Ramadan*, the ninth month of the Muslim lunar calendar, is a unique model of long-term intermittent fasting. In some Islamic countries, *Ramadan* is associated with lifestyle changes that include changes in meal frequency and composition, sleep/wake patterns, and sleep duration [1], [2]. During *Ramadan*, Muslims must refrain from eating and drinking from dawn to sunset. *Ramadan* follows the Islamic (*Hijri*) year that follows the lunar system, which is shorter than the Gregorian year by 11 days. Thus, *Ramadan* occurs during a different season every nine years, which significantly influences the durations of day and night. Furthermore, changes occur in the day/night activity patterns during *Ramadan*, such as late night prayer and the early awakening for the pre-dawn meal (Suhur). Moreover, in some Islamic countries, stores and shopping malls open late and remain open far into the night. Additionally, eating habits may change in some cultures during *Ramadan*; specifically, people prefer fried foods and consume an excessive amount of sweets [1]. The above patterns indicate that the physiological changes that occur during *Ramadan* fasting may be different from those that occur during experimental fasting [3].

Leptin, the protein product of the *obese* gene, is thought to play a role in long-term regulation of caloric intake, fat deposition, and energy balance [4], [5]. It acts as a signal to the brain, reflecting energy stores in the body [6]. The circadian rhythm of serum leptin has previously been demonstrated in normal subjects, with peak averages between 22:00 and 03:00 [7], [8]. Morning leptin levels were relatively high; however, a dramatic reduction in plasma leptin was noted after breakfast, and the lowest level was between 08:00 and 17:00 [8]. Furthermore, it has been shown that plasma leptin profiles were higher in obese than in lean subjects and higher in women than in men [9]. Moreover, meal time shifting has been shown to entrain the diurnal rhythm of leptin, which indicates that eating may be associated with the nocturnal increase in leptin [10]. Ghrelin is an appetite-stimulating peptide that appears to act as a peripheral orexigen that counteracts the action of leptin [11]. Several studies have shown a competitive interaction between ghrelin and leptin in the regulation of food intake [12], [13]. Animal studies have shown that fasting and food intake affect both hormones [14]. In humans, plasma ghrelin levels increase preprandially and decrease after meals [15]. Previous studies have shown that *Ramadan* fasting may influence the circadian rhythms of several biological variables, including hormones [3]. The long-term changes in plasma leptin levels during intermittent *Ramadan* fasting have not been extensively studied. Bogdan et al showed a shift in the circadian rhythm of leptin but no significant changes in the mean concentration of plasma leptin levels on the 23rd day of *Ramadan* daytime fasting in 10 male subjects [16]. By contrast, other studies showed that short-term fasting results in a 30–66% reduction in leptin levels [17]. Prolonged fasting has been shown to increase plasma ghrelin levels in mice and rats [12], [18]. However, the changes in ghrelin plasma levels have not been assessed during *Ramadan* fasting.

Several lifestyle changes that occur during *Ramadan* may influence plasma leptin and ghrelin levels, such as meal time, sleep/wake schedule and sleep duration [19], light exposure [20], and exercise [21]. Previous studies that assessed the levels of leptin during *Ramadan* fasting did not monitor sleep objectively to document sleep duration and sleep quality, did not account for the possibility that attendant cultural and lifestyle changes that occur during *Ramadan*, such as delays in starting school and work, increased activity in malls until late at night, and other changes in the day-night activity patterns that may affect the sleep patterns of individuals regardless of fasting.

Therefore, this study was designed to assess the effect of intermittent Islamic fasting during and outside of *Ramadan* on plasma levels of leptin and ghrelin while controlling for the sleep/wake schedule, sleep duration and quality, meal composition, energy expenditure, and light exposure. Fasting outside of *Ramadan* season aimed to simulate *Ramadan* Islamic intermittent fasting in the absence of the -above mentioned- lifestyle changes that occur during *Ramadan*, which may affect the measured hormones.

## Subjects and Methods

### Subjects

The study protocol was approved by the College of Medicine Institutional Review Board (IRB) at King Saud University. Eight non-smoking, healthy Muslim volunteer males between the ages of 20 and 35 years gave their written informed consent to participate in the study. Volunteers were recruited through advertising in Bulletin Boards in the University. The advertisement clearly stated that volunteers are being recruited for research purposes. The IRB in our institute approved the proposed advertisement. The selected participants were not taking any medications (prescription or over-the-counter), did not drink alcohol, did not perform shift work, and were not on vacation during the study period. None of the participants had sleep complaints, had undergone gastrointestinal surgery, or was on a special diet for medical reasons or to lose weight. To assure a regular sleep/wake schedule before starting the study, all participants were given a wrist actigraphy (Philips/Respironics, Inc., Murrysville, Pennsylvania, USA) for 1 week. None of the participants had travelled during the 2 weeks prior to their enrollment or during the study. Their working hours were from 07:30 to 16:30 before *Ramadan* and from 10:00 to 15:00 during *Ramadan*.

### Study protocol

This study was performed during the last week of *Rajab* (month 7, *Hijri*), the first and last weeks of *Shaban* (month 8, *Hijri*), and the second week of *Ramadan* (month 9, *Hijri*) during the *Hijri* year 1432. The study period corresponded to the dates between June 25 and August 15, 2011, on the Gregorian calendar.

Participants reported to the SDC on four occasions, listed below. During each visit, the subjects spent around 24 hours at the SDC:

1. **Adaptation**: During the last week of *Rajab*, to undergo a medical checkup and to adapt to the environment before the start of the study. Adaptation to the equipment and sleeping environment will serve to avoid the “first night effect”, which may result in alteration of sleep architecture observed on the first night of polysomnographic (PSG) studies [22]. During the adaptation visit, the participants completed the demographic data form and the Epworth Sleepiness Scale (ESS). The ESS is an eight-item questionnaire designed to explore a person's likelihood of falling asleep in common settings [23]. The questionnaire asks the subject to rate his or her probability of falling asleep on a scale of increasing probability from 0 to 3 for eight different situations that most people engage in during their daily life. The ESS is a validated questionnaire used to assess daytime sleepiness [24], [25]. An ESS score of ≥10 was used as an exclusion criterion. All the participants were given an actigraphy monitor to wear on their non-dominant wrist to ensure a regular sleep/wake schedule [26]. A regular sleep/wake schedule was defined as a less than 1 hour daily variability in bedtime and rise time. Body mass index (BMI) was measured during all visits to the SDC.
2. **Baseline fasting (BLF)**: During the first week of the month of *Shaban* (while fasting, baseline fasting) for polysomnography (PSG). This fasting period is not part of the normal routine religious *Ramadan* fasting. This is a protocol we used to simulate *Ramadan* Islamic intermittent fasting outside of *Ramadan* season to control for the lifestyle changes that occur during *Ramadan* and may affect the measured variables. This fasting was performed 3 weeks before *Ramadan* to avoid any overlap with BL or *Ramadan* fasting and any carryover effects.
3. **Baseline (non-fasting) (BL)**: During the last week of the month of *Shaban* (baseline without fasting) for PSG.
4. ***Ramadan***: During the second week of *Ramadan* for PSG.

Participants reported to the SDC at approximately 18:00. The details of the protocol are described in a previous publication [27]. At the SDC, for sleep studies, each participant was given meals with a fixed caloric intake and fixed proportions of carbohydrates, fats, and proteins based on their ideal body weight. During BL, 3 meals were served, namely, dinner at 20:00, breakfast at 07:15, and lunch at 12:00 (mid-day). During BLF and *Ramadan*, three meals were served: breakfast (at sunset, between 18:30 and 18:55), dinner at 21:00, and Suhur before dawn (between 03:00 and 03:15).

At 18:00, an intravenous cannula was placed in the antecubital vein for blood withdrawal. Blood samples were collected at 22:00, 02:00, 04:00, 06:00, and 11:00. Samples were immediately placed on ice, centrifuged at 4 °C, and stored at −70°C until the time of assay.

### Fasting protocol

During baseline fasting, the participants were asked to fast for the first week of *Shaban* (7 days) from dawn to sunset to assess the effects of Islamic intermittent fasting in the absence of the lifestyle changes and eating habits that occur during *Ramadan*. During the subsequent 3 weeks of *Shaban*, the participants practiced their routine activities and eating habits. The participants fasted during the entire month of *Ramadan*. During the baseline fasting (BLF) period, dawn (the beginning of fasting) occurred at approximately 03:37 and sunset (the end of fasting) occurred at approximately 18:47. During *Ramadan*, dawn and sunset occurred at approximately 04:00 and 18:34, respectively.

### Polysomnography

The PSG recordings are detailed in a previous publication [27]. The sleep studies were performed at the SDC. Alice 5 diagnostic equipment (Philips/Respironics, Inc., Murrysville, Pennsylvania, USA) was used for data acquisition. The subjects were asked to avoid napping during the sleep study day. During BL, the study began at 23:00 and was completed at 07:00. During BLF and *Ramadan*, the study began at 2300. During *Ramadan*, the participants were awakened at 03:00 for Suhur, and the study was resumed from 03:45 until 07:45. During BLF, the participants were awakened at 03:15 for Suhur (to account for the shift in dawn prayer time), and the study was resumed from 04:00 until 07:45.

During the study, the following parameters were monitored: four electroencephalography (EEG) channels (C1-A4, C2-A3, O1-A4, and O2-A3), chin electromyography (EMG), electrooculography (EOG) (eye movement), EKG, oxygen saturation, chest and abdominal wall movements, air flow (thermistor), body position, and a microphone for the recording of snoring. “Stage shifts” was defined as total number of changes in sleep state from lights out to lights on. Arousal index, which is a measure of sleep fragmentation, was defined as the number of arousals per 1 hour of sleep.

### Light exposure

The light intensity was measured using a Spectral Star Light Meter LX-1 (Japan). From 18:00 until bedtime and during Suhur, the light level was maintained at 50 lux. During the PSG recordings, all lights were turned off and the light level was maintained at <1 lux.

### Energy expenditure measurement

Energy expenditure while in the SDC was measured using the SenseWear Pro Armband™ (Body Media, Pittsburgh, PA). Technical characteristics of the Armband have been described previously [28], [29]. Briefly, it is a portable sensing device that is 8.8×5.6×2.1 cm in size and 82 g in weight and is worn on the triceps of the right arm. The device provides information regarding the total energy expenditure, total sleep time, and circadian rhythm. The sensors in the Armband measure skin temperature, galvanic skin response, heat flux from the body, and movement. These physiological data are processed by advanced algorithms to calculate and report the total energy expenditure [30]. The algorithm has been validated in many studies and compared with findings obtained using double-labeled water and metabolic carts [29], [31].

### Ghrelin and leptin assays

Collected blood samples were centrifuged at 4°C. The plasma samples were stored at −70°C for subsequent measurement of leptin and ghrelin concentrations. The total blood volume per sample was 5 mL to avoid affecting the ghrelin assay, which requires the highest volume. Plasma leptin levels were determined using enzyme-linked immunoassays (ELIZA; R&D Systems, Minneapolis, MN, USA). Total ghrelin levels were measured by enzyme-linked immunoassays (ELIZA; BioVendor–Laboratorní medicína a.s., Heidelberg, Germany). The ghrelin standard curve was between 1.95 and 250 p/mL. All leptin and ghrelin samples were run in duplicate.

### Statistical analysis

Data are expressed in the text and figures as the mean ± standard error of the mean (SEM). To assess energy expenditure, namely the METs (metabolic equivalents), data were analyzed and averages were calculated to obtain an overall hourly average for each day. We developed a 24-hour cosinor rhythmometry model 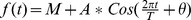, where M = mesor; A = amplitude; Ø = acrophase, and T = 24 hours) to obtain the best estimates of the overall acrophase for leptin and ghrelin [32]. The model was executed for each subject's data during the three periods. Comparisons of the BL, BLF, and *Ramadan* fasting groups were performed using a one-way ANOVA. If the normality test failed, Friedman's ANOVA by rank test was used. Post hoc analyses were performed to determine the significant differences between individual groups. Results were considered statistically significant at the value of p<0.05. Standard statistical software (Sigma Stat, version 3; SPSS, Chicago, IL, USA) was used for the analyses.

## Results

The participants had a mean age of 26.6±4.9 years, a mean BMI of 23.7±3.5 kg/m^2^, and a mean ESS score of 7.3±2.7. There were no changes between periods in BMI. Table 1 shows the sleep pattern at home and sleep parameters in the SDC, as well as energy expenditure during monitoring in the SDC (day and night), during the daytime (from dawn to sunset), and at night (sunset to dawn). There was no difference in energy expenditure during the three monitoring periods (BL, BLF, and *Ramadan*). During the three monitoring periods in the SDC, there was no significant difference in the energy expenditure, TST, sleep efficiency, sleep onset latency, and REM onset latency between the BL, BLF, and *Ramadan* periods. Sleep efficiency was relatively lower than normal (BL = 79.3±9.6, BLF = 70.7±11.1 and *Ramadan* = 74.2±7.3), which could be related to the fact that blood has to be drawn a few times during sleep. Serum glucose measurements at 15:30 revealed no differences between BL, BLF, and *Ramadan* (6.1±0.5, 5.7±0.4, and 5.8±0.5 mmol/L, respectively).

**Table 1:** Sleep pattern while at home and sleep parameters and energy expenditure while under monitoring at the Sleep Disorders Center.

	BL	BLF	*Ramadan*	p-value
Weight (kg)	69.1±8.4	67.5±7.8	66.3±12.3	0.271
**Sleep pattern obtained using actigraphy when sleeping at home**				
Mean Bedtime	00:36	01:30	02:42*	0.004
Mean Wake-up	05:30	06:12	08:48*	0.034
*n*TST (hours)	5.3±1.2	5.3±1.2	5.1±1.3	0.134
*n*TST + NAP (hours)	5.9±1	6.2±2.9	7±2.9	0.539
**Sleep parameters while under monitoring at the SDC using polysomnography**				
Total sleep time (minutes)	375.8±46.4	333.0±59.8	353.9±42.7	0.165
Sleep efficiency	79.3±9.6	70.7±11.1	74.2±7.3	0.336
Sleep latency	29.6±24.2	33.7±33.6	32.8±29.3	0.224
Arousal index	9.1±3.6	8.9±4.8	7.9±3.3	0.369
Stage shifts	66.3±13.9	74.9±20.5	64.1±16.7	0.327
**Energy expenditure based on the SenseWear Pro Armband**				
Energy Expenditure (24 hours) (kcal/minute)	1.6±0.2	1.6±0.2	1.6±0.2	0.846
Energy Expenditure (Daytime) (kcal/minute)	1.7±0.3	1.6±0.2	1.5±0.2	0.069
Energy Expenditure (Night) (kcal/minute)	1.5±0.1	1.6±0.2	1.6±0.2	0.260
METs	1.4±0.2	1.4±0.1	1.3±0.2	0.738
METs (Day)	1.4±0.2	1.3±0.1	1.3±0.1	0.069
METs (Night)	1.3±0.2	1.4±0.1	1.4±0.2	0.311

**Figure 1:**
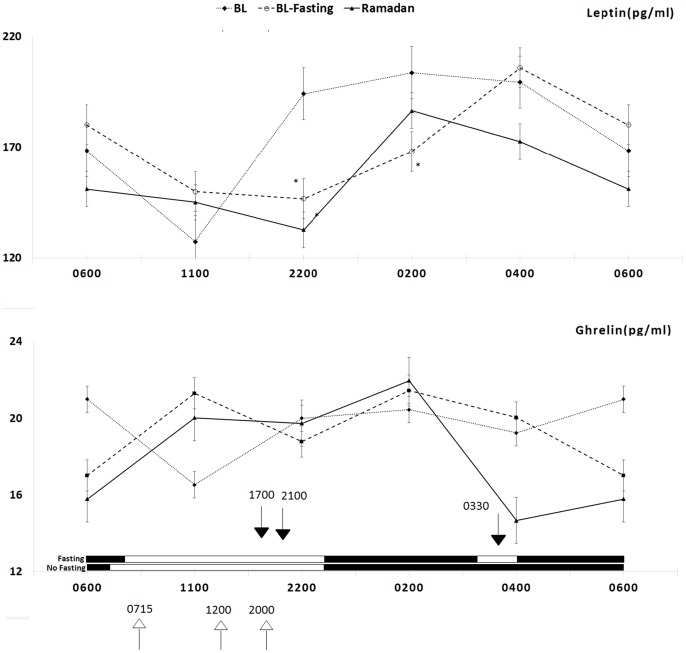
Circadian pattern of serum leptin and ghrelin concentrations in eight healthy male volunteers before and during *Ramadan* daytime fasting. Each point represents the mean and SEM of eight subjects. Values of BL (…♦…), BL fasting before *Ramadan* (---▪---), and *Ramadan* (-▴-) are presented. Dark head arrows indicate meal time during fasting (*Ramadan* and BLF) and white head arrows indicate meal time during BL. White bars indicate awake and black indicates sleep.

Figure 1 shows the pattern of leptin and ghrelin concentrations during BL, BLF, and *Ramadan* fasting. Clearly, the restriction of food and beverage intake to the night hours in BLF resulted in a significant time shift of leptin plasma concentration at 22:00 and 02:00 compared with the baseline (at 22:00: 194.2±177.2 vs. 146.7±174.5; at 02:00: 203.8±189.5 vs. 168.1±178.1; p<0.05). During *Ramadan*, an obvious trend toward a reduction in leptin concentrations at night was clear; however, it was not found to be statistically significant except at 22:00 (194.2±177.2 vs. 132.6±130.4, p<0.05). Similarly, no significant difference in the mean concentration of leptin was found when comparing BL with *Ramadan* fasting and BLF (p>0.05). The data do not show any significant difference in plasma ghrelin concentrations between the three periods. Although the plasma ghrelin concentrations demonstrated a reduction at 04:00 and 06:00 during *Ramadan* fasting compared with BL, the difference was not statistically significant. Plasma ghrelin concentrations were higher at 11:00 during BLF and *Ramadan* compared with BL, but the difference was not statistically significant.

Cosinor analysis of leptin and ghrelin plasma levels (Table 2) revealed no significant changes in the circadian rhythm in the acrophase of the hormones during the three periods.

**Table 2:** Cosinor summary of plasma leptin and ghrelin concentration circadian rhythms during baseline, baseline fasting, and *Ramadan*.

	BL	BLF	Ramadan	p-value
**Ghrelin Circadian Rhythm**				
Mesor	21.7+11.6	19.9+11.7	22.7+8.4	0.607
Amplitude	5.4+2	6.1+4.4	5.5+1.6	0.607
Acrophase	11.7+9.7	11.7+5.6	13.6+7.1	0.607
**Leptin Circadian Rhythm**				
Mesor	121.3+89.8	105.8+82	103.4+75.4	0.180
Amplitude	28.8+26.9	54.7+29.1	25.2+24	0.012
Acrophase	11.1+11.6	7.5+4	8.3+4.8	0.867

## Discussion

Several lifestyle changes that occur during *Ramadan* influence the sleep/wake pattern, sleep duration, physical activity, and meal composition [1], [33]. These changes may influence plasma leptin and ghrelin levels, such as meal time [10], sleep/wake schedule and sleep duration [19], light exposure [20], and exercise [21].To control for potential confounders that accompany the lifestyle changes during *Ramadan*, we assessed the effects of intermittent Islamic fasting outside of *Ramadan*. To our knowledge, no study has assessed the effects of intermittent Islamic fasting on leptin and ghrelin plasma concentrations outside of the *Ramadan* season. This study aimed to assess the changes in the plasma levels of leptin and ghrelin during intermittent fasting while controlling for potential confounders that might affect the levels of both hormones, such as the sleep/wake schedule, sleep duration and quality, physical activity, light exposure, and meal composition. Previous studies have demonstrated an important relationship between sleep and metabolic hormones including leptin and ghrelin. Taheri et al analyzed that data of 1,024 volunteers from the Wisconsin Sleep Cohort Study [19]. The investigators reported that short sleep duration was associated with low leptin with a predicted 15.5% lower leptin for habitual sleep of 5 hours versus 8 hours, and high ghrelin with a predicted 14.9% higher ghrelin for nocturnal sleep of 5 hours versus 8 hours, independent of BMI [19]. Therefore, in the current study, we controlled for sleep duration and sleep/wake schedule to assess the effect of Islamic intermittent fasting on leptin and ghrelin independent of sleep duration. Meal time during fasting periods could not be matched to the baseline because the change in meal time is an essential part of the religious practice during fasting. We intended to assess the effect of intermittent Islamic fasting and the changes in meal time on plasma levels of leptin and ghrelin. The main finding of this study is that the pattern of plasma leptin concentration (Figure 1) demonstrated a significant reduction at 22:00 and 02:00 during BLF and at 22:00 during the second week of *Ramadan* compared with BL. Furthermore, the changes in plasma leptin concentration in this study were not associated with any significant changes in ghrelin plasma concentration. However, when a cosinor model was used, no shift delay was detected in the circadian rhythm of leptin, and ghrelin plasma levels showed no changes during fasting.

The pattern of plasma leptin concentration during BL is in agreement with previously reported data [10], [16]. Limited data are available regarding the effect of prolonged intermittent fasting on plasma leptin levels. The measurements during BLF were performed after one week of fasting, while the measurements during *Ramadan* were performed after 2 weeks of fasting. Bogdan et al, using cosinor analysis, demonstrated a significant shift of approximately 5 hours in the peak and nadir of serum leptin levels on the 23rd day of *Ramadan* fasting [16]. However, they did not find significant changes in the amplitude or 24-hour mean concentration of leptin compared with readings before *Ramadan* [16]. In the present study, cosinor analysis did not reveal changes in the circadian rhythm of leptin. A possible explanation for the difference between our results and those of Bogdan et al is the fact that those authors did not control for behavioral customs such as eating habits and environmental conditions, including light exposure, which might have resulted in a shift delay in the circadian rhythm of the participants [16]. Eliman and Marcus, in an experimental fasting study (fasting for 48 hours), reported a rise in leptin levels after the evening fast-breaking meal [34]. This result is in contrast to that of the present study, which showed a significant reduction in leptin at 22:00. Our findings concur with those of Bogdan et al, who reported no significant increase in the evening levels of leptin [16]. The discrepancy between the Eliman and Marcus study and our study and the Bogdan study could be due to the regional variations in the culture and/or dietary habits, as previously suggested [35], or the longer duration of daytime fasting and the long duration of the practice of fasting, which may modify the body's response [16].

Ghrelin is an endogenous ligand for the growth hormone receptor that was isolated from human and rat stomachs [36]. A preprandial rise in plasma ghrelin levels has been documented, which indicates a role for meal initiation [15]. In this study, we found no significant changes in the ghrelin plasma levels during fasting. To our knowledge, no study has assessed the circadian rhythm changes in plasma ghrelin levels during intermittent Islamic fasting. However, in an experimental fasting study by Natalucci et al, 6 volunteers performed fasting for 33 hours (from 00:00 on day 1 until 00:90 on day 2), and plasma ghrelin levels were measured every 20 minutes for 24 hours (from 08:00 on day 1 until 09:00 on day 2) [37]. They reported a significant decrease in ghrelin levels over a 24-hour period [37]. In another experimental fasting study, Chan et al asked six volunteers to fast for three days, and blood samples were collected every 15 minutes for 24 hours on day 3 [38]. Fasting for three days abolished the meal-related pattern of ghrelin secretion. In addition, they demonstrated that neither the low leptin level during the 72-hour fasting period nor r-metHuLeptin administration in physiological doses increases ghrelin levels compared with those in the baseline state in the same subjects [38]. These findings concur with our findings that ghrelin levels did not increase during fasting and that the reduction in plasma leptin levels was not associated with an increase in ghrelin levels. It has been suggested that the observed meal-related changes in ghrelin levels may be due mainly to post-prandial decreases, rather than preprandial increases, in ghrelin secretion [38]. Several studies have reported a lack of effect of a combination of intravenous glucose and insulin [39], hypoglycemia [40], or hyperglycemia on ghrelin secretion [40], [41]. In addition, distending the stomach with water alone had no effect on ghrelin secretion [42]. However, the consumption of non-caloric fiber resulted in a reduction in ghrelin levels [43]. Mechanisms other than meals may be associated with the control of ghrelin secretion. Natalucci et al demonstrated a characteristic pulsatility of ghrelin secretion and suggested that neuronal rather than gastrointestinal signals may modulate the secretion of ghrelin [37]. Another possible explanation for the unchanged ghrelin level is the fact that sleep duration and pattern did not change in the current study in the three study periods (BL, BLF and *Ramadan*). Because we measured ghrelin and leptin after 1 week of fasting during BLF and after 2 weeks of fasting during *Ramadan*, it is possible that the responses after prolonged fasting are different from those after short-term fasting. Chan et al suggested that tachyphylaxis may occur after prolonged fasting [38]; however, this topic requires further investigation. Future studies should assess the effect of Islamic fasting after a shorter period of fasting like 1 or 2 days on ghrelin and leptin levels.

The fact that the participants were recruited from within a university setting may limit the representativeness of the sample. As this group are more likely to be more educated than the general public.

In conclusion, our investigation on leptin plasma concentration in eight fasting subjects during and before *Ramadan* revealed a nocturnal reduction of leptin that may be a result of the changes in the meal times during fasting. However, cosinor analysis revealed no changes in the circadian rhythm of leptin. Our data showed no change in ghrelin concentration either during or before *Ramadan*.
